# Study on the long-term curative effect of repeat percutaneous balloon mitral valvuloplasty in patients with mitral restenosis

**DOI:** 10.1097/MD.0000000000016790

**Published:** 2019-08-09

**Authors:** Ling Zhang, Jiliu Hou, Yulong Duan, Junjun Chen, Huijuan Du, Zhengang Shi

**Affiliations:** Cardiovaccular Medicine Ward 3, The Second People's Hospital of Pingdingshan, Pingdingshan, China.

**Keywords:** long-term curative effect, mitral restenosis, repeat percutaneous balloon mitral valvuloplasty

## Abstract

To study the long-term curative effect of repeat percutaneous balloon mitral valvuloplasty (PBMV) in patients with mitral restenosis.

In our study, mitral restenosis developed in 39 patients after PBMV. Repeat PBMV was performed according to the improved Inoue method. All patients were followed up.

Of 39 patients, 36 were successfully treated with repeat PBMV (achievement ratio, 92.3%). Immediately after repeat PBMV, clinical symptoms and left atrial mean pressure (LAP), pulmonary artery systolic pressure (PASP), mitral valve gradient (MVG), and mitral valve orifice area (MVA) improved significantly (24.50 ± 6.54 mmHg vs 9.66 ± 4.21 mmHg for LAP, 1.05 ± 0.19 cm^2^ vs 2.23 ± 0.22 cm^2^ for MVA, 17.03 ± 4.52 mmHg vs 7.79 ± 4.07 mmHg for MVG, 58.12 ± 12.68 mmHg vs 31.45 ± 10.02 mmHg for PASP; *P* <.05). Meanwhile, left atrial end-diastolic dimension (LAD) was altered slightly (4.71 ± 0.75 vs 4.07 ± 0.69, *P* >.05). The 36 patients were followed up for 69 ± 23 (12–146) months. After long-term follow-up immediately after repeat PBMV, the results did not show a significant change (2.23 ± −0.22 cm^2^ vs 2.02 ± −0.21 cm^2^ for MVA, 7.79 ± −4.07 mmHg vs 9.15 ± -4.11 mmHg for MVG; *P* >.05) and were approximated to those shortly after repeat PBMV (2.23 ± 0.22 cm^2^ vs 2.02 ± 0.21 cm^2^ for MVA, 7.79 ± 4.07 mmHg vs 9.15 ± 4.11 mmHg for MVG; *P* > 0.05). LAD did not change significantly (4.13 ± 0.71 cm vs. 4.07 ± 0.69 cm; *P* >.05). The long-term follow-up results showed that cardiac function and quality of life were significantly improved in most patients.

It would be safe for patients with mitral restenosis to undergo repeat PBMV. Appropriate cases should be selected, and treatment should be performed cautiously. Short- and long-term curative effects would be satisfactory. We suggested that repeat PBMV be the first choice for patients with mitral restenosis after first PBMV.

## 1 Introduction

Percutaneous balloon mitral valvuloplasty (PBMV) is one of preferred treatment in heart disease intervention therapy in recent years. Since it was reported by Inoue et al in 1984, PBMV has greatly development in rheumatic heart disease mitral stenosis (MS).^[1–4]^ Decrease in the incidence of rheumatic heart disease in developed countries had already begun in 1910, and it is now below 1.0 per 100,000. In contrast, the occurrence rate of rheumatic heart disease in developing countries remains substantial.^[5]^ The mechanism of PBMV is the same as the already abandoned closed mitral commissurotomy.^[6]^ Pathological studies have disclosed that the main mechanism of successful PBMV is a fracture of the commissures. Compared to surgical mitral commissurotomy, PBMV has shown equal or better^[7,8]^ success rates and comparable restenosis rates. A total of 1138 consecutive PBMVs were retrospectively reviewed. These procedures were performed between 1988 and 2004 using the PBMV technique. Of the PBMVs, 35 repeat PBMVs were identified between 1989 and 2012.^[9]^ Incidence of MS in PBMV increased annually, and about 7% to 21% of patients showed symptomatic restenosis.^[10]^ In recent years and with literature on repeat balloon angioplasty for restenosis after PBMV growing rapidly, the short-term treatment effect after PBMV with Wilkins score of 8 or 9 to 11^[11]^ is better.^[12]^

Although more studies focus on MS after PBMV and repeat PBMV,^[2,13–15]^ fewer studies report on the long-term curative effect of repeat PBMV, which is an important research in treatment and prognosis. Therefore, in this study, based on long-term clinical practice, we reported the long-term curative effect of repeated PBMV in 39 patients.

## 2 Materials and methods

### 2.1 Patients

Patients were included in this study according to the following criteria:

1) Patients had MS and were treated with PBMV according to the Management of Valvular Heart Disease of the European Society of Cardiology and the European Association for Cardio-Thoracic Surgery.^[16]^
2) After successful PBMV, patients had decreased mitral valve area (MVA, ≤1.5 cm^2^) or >50% decrease in MVA during the follow-up process and also had diverse symptoms with different levels.^[2]^
3) Integrals of ultrasound mitral valve morphology, using the Wilkins method, were <10.^[11]^
4) NYHA class of cardiac function was II to IV.
5) Patients with moderate-severe mitral incompetence (MI) and/or moderate-severe aortic valve disease were excluded from this study.

We evaluated patients with MS who were treated in our hospital from October 1988 to October 2006. The patients were examined by transthoracic echocardiography using the Acuson Sequoia C256 equipped with a 15-MHz linear transducer (Sequoia C256; Acuson, Mountain View, California). Echocardiographic measurements were obtained using an Ekoline 20A ultrasound receiver interfaced with a Honeywell 1856 Linescan Recorder. A 2.25-MHz 1.25-cm-diameter Aerotech ultrasonic transducer was used. The echography operators were the same in the study. The morphology of the cardiac mitral valve was assessed by Wilkins ultrasonography. In the score system, the degree of valvular activity, valvular thickening, subvalvular lesion, and valvular calcification were divided into 4 morphological lesions of the mitral valve by echocardiography, each of which was scored with 1 to 4 points, with a total score of 0 to 16 points. The higher the score, the heavier the mitral valve lesion. Of these, 13 patients were found to have recurrent stenosis after primary PBMV in other hospitals, 35 patients (74.3%) were found to have recurrent stenosis after primary PBMV in the follow-up visit in our hospital with significant clinical symptoms, and 9 patients did not have significant symptoms and were not treated with repeat PBMV. Therefore, a total of 39 patients were treated with repeat PBMV, and of these, PBMV was successful in 36 (92.3%). The average age of these 36 patients (11 men and 25 women) was 31.6 ± 9.3 years (21–52), and the average duration of rheumatic disease was 16.9 ± 9.8 years (8–35). Sinus rhythm was detected in 21 patients, and continuous or perpetual atrial fibrillation (AF) in 15 patients. AF development alone may be an indication of PBMV. In the NYHA classification of cardiac function was class II in 3 patients (8.4%), class III in 25 patients (69.4%), and class IV in 8 patients (22.2%). This study was conducted in accordance with the declaration of Helsinki. This study was conducted with approval from the Ethics Committee of The Second People's Hospital of Pingdingshan. Written informed consent was obtained from all participants.

### 2.2 PBMV operative method

The improved Inoue single-balloon technique was used to perform PBMV.^[17]^ Venous cannulas were inserted from the femoral vein, and room puncture was intersected at the point of the left atrium, lower 1/3 junction line, and spine, and right 1/3 junction vertical line was intersected under X-ray perspective after successful puncture. During the process of withdrawing the puncture needle, from the top to bottom of its outer sheath, a specific sensation should be noted on the puncture site, and puncture was performed when there was a “push” sensation. Based on left atrial pressure curve monitoring and contrast agent “smoke” test, puncture was ensured when the left atrium was penetrated. After successful puncture, 3000-μ heparin was injected via the catheter. A balloon catheter was fed into the left atrium, and its position and direction were adjusted to ensure that the tip penetrated the left atrium via the mitral orifice. Our improved method used a small radian when pushing the balloon catheter over the mitral valve to penetrate the left atrium via the mitral orifice. Wilkins scores were recorded according to the heights of patients, which were used to select the effective diameter of balloon expansion. Rapid inflation after withdrawal of the balloon in the mitral valve promoted complete extension. The size of the sacculus was estimated using the maximum diameter based on the following formula: (height [cm])/10 + 10, and then 2 to 3 cm is subtracted to the original extended diameter. Then, according to the cardiac function, valve condition, and lesion level, the balloon diameter was increased using the balloon diameter increment method; the largest extended diameter was 22 to 28 mm from 20 mm with gradually increase of 0.5 to 1 mm.

### 2.3 Determination of effective repeat PBMV

Echocardiographic examinations were preformed pre- and postoperatively (1–2 months) to measure the left atrial diameter and MVA, and determination of the MVA was based on 2-dimensional echocardiography. These were estimated using half of the stress time. In PBMV, the average left atrial pressure, cross-valve pressure difference, and pulmonary systolic pressure were measured through pressure monitoring. All statistical data were reordered using echocardiographic measurements. The determination criteria of a successful repeat PBMV were as follows: complete operation process, increase in MVA by >50% or MVA >1.5 cm^2^ postoperatively, absence of severe MI, significantly reduced or absent apical diastolic murmur.

### 2.4 Follow-up

The average follow-up period was 69 ± 23 (12–146) months after successful repeat PBMV in 36 patients. The main follow-up data mainly included symptom, sign, NYHA classification, electrocardiography, and 2-dimensional and Doppler echocardiography. Each patient was followed every 3 months, and the last follow-up findings were considered the final results.

### 2.5 Statistical analysis

All measurement data were described as 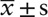 (mean ± standard deviation). The *t* test was used to compare preoperative and postoperative follow-up data, and the x^2^ test was used to estimate the potential difference in enumerated data.

## 3 Results

### 3.1 Short-term observation of curative effect of repeat PBMV

Of 39 patients, 36 were successfully treated with PBMV, and the success rate was 92.3%. The procedure failed in 3 patients, including 1 patient with room puncture, 1 patient with acute pericardial tamponade, and 1 patient with severe MI. After successful PBMV, symptoms, such as chest tightness and shortness of breath, were significantly reduced; heart sound of the pulmonary valve area second was reduced than that pre-operatively; apical diastolic murmur significantly decreased or disappeared; and systolic murmur also reduced or unchanged. Significant differences in MVA, mean left atrial pressure, transvalvular pressure gradient, and pulmonary arterial systolic pressure were noted pre- and postoperatively but left atrial diameter did not have a significant difference (Table 1).

**Table 1:**
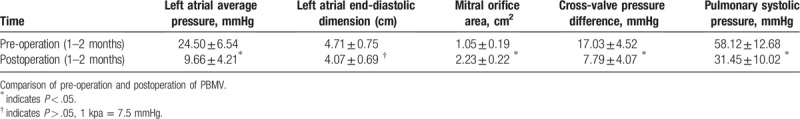
Short-term curative effects in patients with repeated percutaneous balloon mitral valvuloplasty 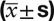.

### 3.2 Long-term observation of curative effect of repeat PBMV

In 36 patients, the average follow-up period was 69 ± 23 months (from 12 to 146 months) after repeat PBMV. We found that MVA was more than that preoperatively, and cross-valve pressure difference was lower and similar to the results of the short-term follow-up. No significant difference in left atrial diameter was noted before and after repeat PBMV (Table 2). Clinical symptoms and quality of life significantly improved in most patients, and cardiac function was categorized as NYHA class I with long-term stability in 21 patients, NYHA class II in 12 patients, and NYHA class III in 3 patients. During the follow-up process, 2 patients had recurrent MS, 1 patient was treated with a third successful PBMV, and another patient was treated successfully with mitral valve replacement. One patient died during follow-up process due to other causes. Balloon dilatation, hemodynamic index, cardiac ultrasonography, clinical symptom, and quality of life were significantly improved.

**Table 2:**
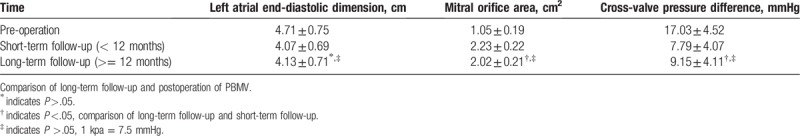
Long-term curative effects in patients with repeated percutaneous balloon mitral valvuloplasty 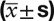.

## 4 Discussion

PBMV was first reported by Inoue in 1984 and is a novel technique to treat MS of rheumatic heart disease.^[4]^ The treatment mechanism is similar to closed mitral commissurotomy: adhesive mitral valve junction splits and enlarges the MVA under a high-pressure balloon. PBMV has been widely performed in the treatment of MS because of its simple process, reliable curative effect, lesser surgical trauma, and fewer symptoms and is an ideal alternative method in the clinic. According to Wilkins scores and presence of mitral valve lesions, 4 factors, including leaf activity, presence or absence of valve thickening and its degree, subvalvular lesions, and valvular calcification, are categorized into 4 grades: 1 to 4 according to the severity, and the total scores are 0 to 16. PBMV with a score of <8 shows better curative effect with lower incidence of complications; PBMV with a score of 9 to 12 shows that satisfactory curative effect was noted in some patients and they can be treated with PBMV; and valve replacement can be performed if the curative effect is poor. PBMV with a score >12 shows poor curative effect with more complications, and valve replacement should be performed as an alternative to PBMV. Patients with PBMV may develop stenosis again, and the main factors primarily include ultrasound mitral morphological integration, age, immediate postoperative effect, and active rheumatism.^[18]^ Of these, active rheumatism is the main factor.

Recently, studies have shown that some patients have gradual mitral valve restenosis and MVA is lower than that preoperatively during long-term follow-up.^[19,20]^ However, cardiac function in some patients are still normal, and they can engage in heavier physical activity with less influence. In our study, after successful primary PBMV, recurrent restenosis was detected in 35 patients, but 9 of them (25.7%) did not have significant clinical symptoms and have normal cardiac function; thus, repeat PBMV was not performed. The main reason may be that cardiac function is affected by integrated factors postoperatively. According to Gorlin formula, CO=3.77 × MVA × T × HR × MVG (CO indicates cardiac output, T indicates left ventricular diastolic filling time, HR indicates heart rate, and MVG indicates mitral valve transvalvular pressure difference), mitral reserve function is the main factor in determining cardiac output increase in equal load status. According to the physiological needs, increase in MVA presents the mitral reserve function. Generally, a normal person's mitral valve reserve rate is 30% to 50%. Therefore, compared with MVA in the resting state postoperatively, mitral valve reserve function is valuable in the estimation of cardiac function. We found that MVA in the resting state is considered as the main index to estimate whether patient with restenosis after PBMV or closed surgical separation should be treated with a repeat operation, but mitral reserve function is the main judgment indicator. Patients with restenosis with some indexes, such as MVA <1.5 cm^2^ and better mitral valve reserve function without significant clinical symptoms, can be further observed.

Patients with restenosis after PBMV or closed surgical separation can be treated by repeat PBMV, which has similar curative effect with primary PBMV and better improvement of symptoms with long-term follow-up.^[21]^ Due to some advantages of PBMV, including higher security, less trauma, and repeatability, it is the primary effective treatment for patients with restenosis with normal valve condition.^[15,22]^ Herein, no significant difference is noted although patients who underwent repeat PBMV have improved left atrial diameter in the short-term and long-term follow-up postoperatively, which may be related to the small sample size in this study. Generally, older patients have a risk of coronary disease due to more severe MS, poor heart function, AF risks, high ultrasound scores, and often other associated valve lesions, and negative correlation exists between age and PBMV effect. In our study, the average age is 31.6 ± 9.3 years (21–52), and immediate curative effect, follow-up valve area, and cardiac function are good. We believe that PBMV may have better curative effect based on good preoperative preparation and careful performance of the operation.

Our study indicates that

1) the curative effect of repeat PBMV mainly depends on mitral valve condition.^[23]^ Most patients have a long medical history, primary PBMV has different levels of damage on the valve, and organization repair process may lead to thickening, adhesion, fusion, and thickening of the subvalvular structure at the junction of leaflets. These may damage the valve. In order to avoid reexpansion that causes valve complications such as tearing, patients should be specifically screened according to valve condition using diverse examinations.
2) The curative effect should be determined by a combination of various methods, including cardiac auscultation, left atrial manometry, and balloon size measurement from the left ventricle to the left atrium, and gradual incremental expansion should be used. The most important point is security, and expansion should be discontinued.
3) For patients with repeat PBMV, room divider may be changed with different levels due to longer duration, higher mean left atrial pressure, and larger left atrium and may be thickened and toughened. Therefore, room puncture is more difficult to perform than primary PBMV, and care should be taken during the operation. Here, after successful repeat PBMV, the valve area enlarges, preoperative and postoperative average left atrial pressures are significantly reduced, and clinical symptoms and quality of life are significantly improved. Primary or repeated operation has a close relationship with ultrasound scores and clinical results, and morphological characteristics of the valve determine the prognosis.

Left atrial thrombosis incidence is up to 30% in patients with rheumatic heart disease and AF;^[24]^ therefore, AF is considered as a relative contraindication of PBMV, when there is a left atrial thrombus active. The intracardiac catheter and guidewire are the main reasons for detachment of left atrial thrombus, leading to systemic embolism. Detection of left atrial thrombus preoperatively and during treatment and heparin use is crucial to prevent systemic embolism. There were several research studies on the long-term outcomes of percutaneous mitral valvuloplasty. A study with a follow-up period of up to 20 years showed that an echocardiographic score >8 and post-MVA ≤1.76 cm^2^ were independent predictors of poor long-term clinical outcomes after PBMV.^[25]^ Another study evaluating the immediate results of repeat PBMV and introducing a simplified redo-score to predict the success of repeat PBMV showed that the redo-score system was significantly more predictive than the Wilkins score and was particularly valuable in predicting outcome in patients who underwent PBMV.^[26]^ We used the following methods to prevent systemic embolism:

1) Patients with AF and/or left atrial thrombosis who are treated with oral warfarin for more than 3 months should receive full anticoagulant therapy.
2) Our improved method uses a small radian when pushing the balloon catheter over the mitral valve orifice, which can prevent the catheter from touching the thrombus formation site at the bottom of the left atrium and the left atrial appendage to reduce embolism.
3) Patients with inadequate anticoagulation must be determined by esophageal ultrasound, and PBMV can be used after full anticoagulation therapy.

The main limitation is the small sample size of essentially 36 and that patients with postoperative restenosis after PBMV should be more cautious in their choice of secondary surgery.

## 5 Conclusion

Altogether, for patients with restenosis after PBMV, repeat PBMV can obtain better hemodynamic changes and clinical efficacy if the correct operation is properly selected for patients. Furthermore, the long-term curative effect of repeat PBMV is better. Therefore, we suggest that repeat PBMV is beneficial to patients with restenosis after initial PBMV.

## Author contributions

**Conceptualization:** Ling Zhang, Huijuan Du.

**Data curation:** Jiliu Hou, Yulong Duan.

**Formal analysis:** Jiliu Hou, Yulong Duan, Huijuan Du.

**Funding acquisition:** Ling Zhang, Jiliu Hou.

**Investigation:** Yulong Duan, Junjun Chen.

**Methodology:** Jiliu Hou, Yulong Duan, Junjun Chen.

**Project administration:** Ling Zhang.

**Resources:** Ling Zhang, Huijuan Du.

**Software:** Junjun Chen, Huijuan Du.

**Supervision:** Huijuan Du, Zhengang Shi.

**Validation:** Junjun Chen, Zhengang Shi.

**Visualization:** Zhengang Shi.

**Writing – original draft:** Ling Zhang.

**Writing – review & editing:** Ling Zhang.
